# The measurement of amylase in drain fluid for the detection of pancreatic fistula after gastric cancer surgery: an interim analysis

**DOI:** 10.1186/s12957-014-0428-y

**Published:** 2015-02-19

**Authors:** Angelo De Sol, Roberto Cirocchi, Micol Sole Di Patrizi, Andrea Boccolini, Ivan Barillaro, Alban Cacurri, Veronica Grassi, Alessia Corsi, Claudio Renzi, Daniele Giuliani, Marco Coccetta, Nicola Avenia

**Affiliations:** Department of General Surgery, Terni Saint Mary Hospital, University of Perugia, Via Tristano di Joannuccio, 1, 05100 Terni, Italy; Division of General and Oncological Surgery, University of Perugia, Località Sant’Andrea delle Fratte, 1, 06134 Perugia, Italy

**Keywords:** Pancreatic fistula, Gastrectomy, Risk factor, Amylase drainage concentration

## Abstract

**Background:**

Pancreatic fistula is still one of the most serious and potential complications after D2-D3 distal and total gastrectomy (4% to 6%). Despite their importance, pancreatic fistulas still have not been uniformly defined. Amylase concentration of the drainage fluid after surgery for gastric cancer can be considered as a predictive factor of the presence of pancreatic fistula.

**Methods:**

From January 2009 to April 2013, 53 patients underwent surgery for gastric cancer. Amylase concentration in the drainage fluid was measured on the first postoperative day and if it was ≥1,000 UI, it was measured again on the third postoperative day. Pancreatic fistula occurred in four cases (7.5%). Pancreatic fistulas were classified using the International Study Group on Pancreatic Fistula (ISGPF) criteria into different grades of severity. Two fistulas were Grade A, one was Grade B, and one was Grade C.

**Results:**

Management of drainage tubes is still crucial after gastrectomy, not only for the likelihood of anastomotic leaks but also the eventual diagnosis and management of pancreatic fistula. High amylase drainage content and then the presence of the pancreatic fistula may be due to several causes: the operation itself when it includes splenectomy or pancreatic tail-splenectomy, the extended lymphadenectomy but even the ‘gently and softly’ pancreatic manipulation, according literature, may be a risk factor.

**Conclusions:**

The authors assessed amylase concentration in the drainage fluid collected from the left subphrenic cavity on POD1 and POD3 in 53 patients who had undergone curative gastrectomy for cancer and concluded that amylase drainage content >3 times the serum amylase was a useful predictive risk factor for pancreatic fistula. Our work is an interim analysis and the aim of this study is to increase the accrual of the number of patients to have a significant number. For this reason, a protocol for a multicenter trial will be designed to verify whether the systematic measurement of amylase in drain fluid is better than abdominal ultrasound for the detection of pancreatic fistula after gastric cancer surgery.

## Background

Pancreatic fistula is still one of the most serious potential complications after D2-D3 distal and total gastrectomy (4% to 6%) [1-4].

Systematic lymphadenectomy, splenectomy, and distal pancreasectomy during the surgical procedure for gastric cancer appear to be responsible for several complications: abdominal abscess, anastomotic leakage, wound abscess, lymphorrhea, anastomotic stenosis, postoperative bleeding, cardiac failure, bowel obstruction and pancreas-related complications such as pancreatic leakage and fistula [2,5] (Table 1).

**Table 1:** **Incidence of postoperative complications after gastric surgery**

**Postoperative complication**	**Incidence (%)**
Wound abscess	7.3
Pneumonia	6
Pancreas-related and abdominal abscess	3-4
Anastomotic leakage	2-6
Lymphorrhea	1.5
Anastomotic stenosis	1.5
Postoperative bleeding	1-3
Bowel obstruction	1.5
Cardiac failure	1

The insertion of drainage tubes can be useful for the prediction and management of these complications. Despite their importance, pancreatic fistulas still have not been uniformly defined.

Amylase concentration of the drainage fluid after surgery for gastric cancer can be considered a useful predictive risk factor for pancreatic-related complications [4-6].

In 2005 the International Study Group on Pancreatic Fistula (ISGPF) developed a universal definition for pancreatic fistula: drain output of any measurable volume of fluid on or after postoperative day 3 with an amylase content >3 times the serum amylase activity [4].

We measured amylase drain fluid concentration and also the volume produced; and we considered this valid only for drain fluid production higher than 400 cc [1].

Many authors consider amylase concentration ≥1,000 UI on the first postoperative day as a significant risk factor for pancreatic fistula.

In this paper we want to demonstrate if amylase concentration in drainage fluid on the first day after surgery for gastric cancer can be considered a useful and potential risk factor for pancreatic-related complications, especially for pancreatic fistula [4,5]. Our study is an interim analysis and the aim of this paper is to increase the number of patients in order to have a significant number.

## Methods

From January 2009 to April 2013, 53 patients underwent surgery for gastric cancer at the Department of General Surgery, Terni Saint Mary Hospital, University of Perugia.

There were 28 men and 25 women, and the mean age of patients was 72.3 years (age range, 42 to 88 years), the patients had given consents.

D2 distal gastrectomy was performed in 30 cases, the remaining 23 had undergone D2 total gastrectomy, including nine cases with splenectomy and one case with pancreatic tail-splenectomy.

The histologic types were: three patients were T1b and three patients were T2, while 27 patients were T3 and 20 patients were T4.

Two drainage tubes connected to a bag for drainage fluid collection were placed in the left subphrenic cavity and Winslow’s cavity in the patient who had undergone total gastrectomy and only one in the left subphrenic cavity in patients treated with D2 distal gastrectomy.

Amylase concentration in the drainage fluid was measured on the first postoperative day and if it was ≥1,000 UI, it was measured again on the third postoperative day. Drainage fluid was sampled from the left subphrenic cavity in all the cases on days 1[5] and 3. We also sampled serum amylase and lipase concentration and tube drainage fluid volume in all of the patients [6].

We removed the drainage tube on postoperative day 7, after radiological control, if the drain amylase concentration was three times lower than serum amylase concentration. We did not remove the drain tube in patients that had drainage amylase value three times higher than serum amylase. (Ethics Committee approval was not required in these cases, because placement of the drainage tubes connected to a bag for drainage fluid collection is common clinical practice).

We define pancreatic fistula when the amylase in drain production is >1,000 IU/L, independent of the volume produced (Tables 2 and 3).

**Table 2:** **Characteristics of the 53 patients enrolled in our study**

**Mean age (years)**	**72.3 (range, 42–88)**
M:F ratio	28:25
TNM	T1-2: 6 patients, T3-4: 47 patients

**Table 3:** **Results of our study: analysis of amylase in abdominal drainage**

**Drainage amylase on postoperative day 1**	**Patients (n)**	**Pancreatic fistula**
<1,000	45	/
1,000-2,000	3	1
2,000-4,000	1	1
>4,000	3	2
Total	53	4

## Results

Postoperative pancreatic fistula occurred in four cases (7.5%) (Table 4). These pancreatic fistulas were classified using ISGPF criteria [4] in different grades of severity (Table 5). Using the ISGPF classification [4], patients in our study with drainage amylase on or after the the third postoperative day three times higher than the normal serum amylase value can be defined in this way: two were Grade A, one was Grade B, and one was Grade C.

**Table 4:** **Description of patients with pancreatic fistula**

**Sex**	**Age (years)**	**Exams**	**Surgery**	**Histology**	**Clevien-Dindo**
M	72	• Endoscopy	TG	Adenocarcinoma of gastric remnant	
		• CT scan	D2 lymphadenectomy	T3 N3 MX R0	Grade IIIa
		• Endoscopic US		G3	
F	81	• Endoscopy	DG		
		• CT scan	D2 lymphadenectomy	T3 N0 MX R0	Grade I
		• Endoscopic US		G2	
M	65	• Endoscopy	DG		
		• CT scan	D2 lymphadenectomy	T1b N0 MX R0	Grade IIIb
		• Endoscopic US		G1	
M	78	• Endoscopy	DG		Grade IIIa
		• CT scan	D2 lymphadenectomy	T2 N0 MX	
		• Endoscopic US		G2-G3

**Table 5:** **ISGPF classification for pancreatic fistula**

No fistula	Drainage amylase on or after postoperative day 3 is not three times than upper normal serum amylase value
Grade A	No specific treatment was required even though drainage amylase on or after postoperative day 3 is three times than upper normal serum amylase value
Grade B	Requires a change management or adjustment of clinical pathway (antibiotics, total parenteral nutrition, or repositioning of drainage tubes)
Grade C	Requires major charge in the clinical pathway; clinical intervention is aggressive and often in the ICU setting

The Grade A patients did not undergo any treatment but they required a longer hospitalization (7 to 10 days longer than normal).

The Grade B patient underwent total parenteral nutrition and somatostatine and then antibiotic treatment (piperacilline + tazobactam 4.5 mg three times a day).

The Grade C patient underwent a second operation for acute pancreatitis, pancreatic abscess, and occlusion for adynamic ileum. This patient underwent abscess drainage and ileostomy. The patient died on the 10th postoperative day due to multiorgan failure.

For patients with a fistula of Grade B or higher, a severity classification of Clavien-Dindo [4] was used (Table 6). Of our four patients with postoperative pancreatic fistula one of them was Clavien-Dindo Grade I, two were Grade IIIa, and one Grade IIIb. The first one (Grade I) was treated only pharmacologically, the other three (IIIa and IIIb) underwent invasive procedures.

**Table 6:** **Clavien-Dindo classification**

**Grades**	**Definition**
Grade I	Any deviation from the normal postoperative course without the need for pharmacological treatment or surgical, endoscopic, and radiological interventions
	Allowed therapeutic regimens are: drugs as antiemetics, antipyretics, analgetics, diuretics and electrolytes, and physiotherapy. This grade also includes wound infections opened at the bedside
Grade II	Requiring pharmacological treatment with drugs other than such allowed for Grade I complications. Blood transfusions and total parenteral nutrition are also included
Grade III	Requiring surgical, endoscopic, or radiological intervention
Grade IIIa	Intervention not under general anesthesia
Grade IIIb	Intervention under general anesthesia
Grade IV	Life-threatening complication (including CNS complications: brain hemorrhage, ischemic stroke, subarachnoid bleeding, but excluding transient ischemic attacks) requiring IC/ICU management
Grade IVa	Single organ dysfunction (including dialysis)
Grade IVb:	Multi-organ dysfunction
Grade V	Death of the patient
Suffix ‘d’	If the patient suffers from a complication at the time of discharge, the suffix ‘d’ (for ‘disability’) is added to the respective grade of complication. This label indicates the need for a follow-up to fully evaluate the complication

Among the patients that developed pancreatic fistula, one underwent total gastrectomy and D2 lymphadenectomy and the other three underwent distal gastrectomy and D2 lymphadenectomy. Two of them were T3, one was T2, and the last one was T1b. Only one T3 patient was N3. According to Lauren histological classification, three were intestinal histotype and one was diffuse histotype. All of them were adenocarcinomas.

No development of pancreatic fistula was observed with less than three times increased amylase during the first 3 postoperative days and absence of pancreatic fistula with more than three times increased amylase during the first 3 postoperative days.

## Discussion

Management of drainage tubes is still very important after gastrectomy [3], not only for the possible anastomotic leaks but also for the prediction and management of pancreatic fistula.

In fact many studies have demonstrated that qualitative analysis of the drainage content is very important to predict and manage this complication after gastric surgery for cancer.

In our discussion, it is important to underline that differential diagnosis between lymphorrhea and pancreatic fistula has been performed measuring triglycerides in drainage fluid.

Many authors [6] consider some parameters:

- drainage amylase concentration
- lipase drainage concentration
- drainage volume.

In our experience, and in accordance with many studies [6], we prefer to consider amylase drainage concentration [5] overall because it has demonstrated that lipase concentration is related to amylase concentration and drainage volume is not significant. It also demonstrated that drainage volume is often elevated in patients with advanced cancer (T3-T4) or those who underwent extended lymphadenectomy [2,6].

Examination of drainage fluid in these patients was normal so we can suppose it was only lymphorrhea [6,7].

High amylase drainage content followed by pancreatic fistula risk may be due to many causes: the operation itself, when it includes splenectomy or pancreatic tail-splenectomy; the extended lymphadenectomy; and even the ‘gently and softly’ pancreatic manipulation.

In conclusion, we affirm that patients who had undergone total gastrectomy with splenectomy and extended lymphadenectomy (D2-D3) had more frequently an amylase content >3 times than the serum amylase concentration on the first and third postoperative days [4].

In fact we can consider the followings as risk factors for pancreatic fistula after surgery for gastric cancer:

- pancreatic manipulation (even gently and softly)
- surgery (pancreatic tail resection, extended lymphadenectomy).

In our experience we noticed that the amylase drainage level was related to the duration of the fistula. Our preliminary data are similar to those in international literature [1,5,8-10] (Table 7).

**Table 7:** **Pancreatic fistula incidence: review of literature**

**Study**	**Surgical treatment**	**Pancreatic fistula incidence (%)**
Iwata *el al.* [5]	D1-D2 gastrectomy	16.3
Tomimaru *et al.* [8]	D1-D2 gastrectomy	9.2
Sano *et al.* [9]	D1-D2 gastrectomy	13.7
Kodera *et al.* [10]	D2-D3 gastrectomy	5.7
Sano *et al.* [1]	D2 gastrectomy	5.3
Sasako *et al.* [11]	Distal and Total gastrectomy	6.0
Furukawa *et al.* [12]	D2 gastrectomy	13
Nobouka *et al.* [13]	D2 gastrectomy	18

## Conclusion

Despite its importance, pancreatic fistulas have still not been uniformly defined. Only a few studies have attempted to correlate the concentration of amylase in drain fluid with the risk of developing complications.

Pancreatic fistula is still one of the most serious complications after D2-D3 gastrectomy for gastric cancer. It is natural that pancreatic fistula is frequent if drain amylase is high. The absolute value of drain amylase has no significance since the value may be changed by ascitic concentrations.

Pancreatic fistula developed in four of our patients and amylase concentration of the drainage fluid seemed to be a useful indicator for this complication in patients with lymphorrhea resulting in D2 gastrectomy. Ascites from lymphadenectomy does not reduce the sensitivity of amylase concentration of the drainage.

In this interim analysis the number of the patients with D2 gastrectomy (53 patients) is too small to reach a definitive conclusion. The future aim of this work is to increase the accrual of the number of patients to have a significant number.

For this reason, a protocol for a multicenter trial will been designed to verify whether the systematic measurement of amylase in drain fluid is better than abdominal ultrasound for the detection of pancreatic fistula after gastric cancer surgery.
